# Mendelian randomization analyses of smoking and Alzheimer’s disease in Chinese and Japanese populations

**DOI:** 10.3389/fnagi.2023.1157051

**Published:** 2023-05-12

**Authors:** Yuan Zhu, Ying Guan, Xuewen Xiao, Bin Jiao, Xinxin Liao, Hui Zhou, Xixi Liu, Feiyan Qi, Qiyuan Peng, Lu Zhou, Tianyan Xu, Qijie Yang, Sizhe Zhang, Meng Li, Zhouhai Zhu, Sheming Lu, Jinchen Li, Beisha Tang, Lu Shen, Jianhua Yao, Yafang Zhou

**Affiliations:** ^1^Department of Neurology, Xiangya Hospital, Central South University, Changsha, China; ^2^Joint Institute of Tobacco and Health, Kunming, Yunnan, China; ^3^Engineering Research Center of Hunan Province in Cognitive Impairment Disorders, Central South University, Changsha, China; ^4^National Clinical Research Center for Geriatric Disorders, Xiangya Hospital, Central South University, Changsha, China; ^5^Department of Geriatrics, Xiangya Hospital, Central South University, Changsha, China; ^6^Bioinformatics Center & National Clinical Research Center for Geriatric Disorders, Xiangya Hospital, Central South University, Changsha, China

**Keywords:** Alzheimer’s disease, smoking, two-sample Mendelian randomization analysis, causal association, East Asian population

## Abstract

**Background:**

Previous epidemiological studies have reported controversial results on the relationship between smoking and Alzheimer’s disease (AD). Therefore, we sought to assess the association using Mendelian randomization (MR) analysis.

**Methods:**

We used single nucleotide polymorphisms (SNPs) associated with smoking quantity (cigarettes per day, CPD) from genome-wide association studies (GWAS) of Japanese population as instrumental variables, then we performed two-sample MR analysis to investigate the association between smoking and AD in a Chinese cohort (1,000 AD cases and 500 controls) and a Japanese cohort (3,962 AD cases and 4,074 controls), respectively.

**Results:**

Genetically higher smoking quantity showed no statistical causal association with AD risk (the inverse variance weighted (IVW) estimate in the Chinese cohort: odds ratio (OR) = 0.510, 95% confidence interval (CI) = 0.149–1.744, *p* = 0.284; IVW estimate in the Japanese cohort: OR = 1.170, 95% confidence interval CI = 0.790–1.734, *p* = 0.434).

**Conclusion:**

This MR study, for the first time in Chinese and Japanese populations, found no significant association between smoking and AD.

## 1. Introduction

As the population ageing, dementia becomes one of the most daunting global health challenges of the 21st century. Global prevalence estimation of dementia found ageing contributing most to projected case increases in East Asia (Collaborators GBDDF, 2022). The most common cause of dementia is Alzheimer’s disease (AD), accounting for about 60–80% of cases (Alzheimers Dement, 2020). In China, it is expected that the number of AD patients will reach about 18 million in 2030 (Jia et al., 2018). In Japan, the prevalence of dementia in people older than 65 years is expected to exceed 25% by 2045 (Nakahori et al., 2021).

The etiology of AD is not well understood, it is well-recognized that both environmental and genetic factors contribute to AD (Scheltens et al., 2021). A large systematic review proposed smoking as 1 of the 9 risk factors with AD (Yu et al., 2020), whereas some prospective cohort studies showed no association between smoking and dementia (Abner et al., 2019; Otuyama et al., 2020). After considering the confounding factors including the competitive risk of death in dementia-free people, smoking was no longer associated with AD neuropathology at autopsy in the follow-up study of 531 cognitively healthy individuals for 11.5 years (Abner et al., 2019). Besides, cognitive enhancing effects of nicotine may increase the difficulty in quitting smoking, especially in smokers with cognitive prior deficits (Valentine and Sofuoglu, 2018; Gungen et al., 2022). Moreover, the specific receptors of nicotine, nicotinic acetylcholine receptors (nAChRs), including *α*7 and *β*2 subunits are enriched in the hippocampus and prefrontal cortex, and modulating a variety of cognitive functions (Valentine and Sofuoglu, 2018). Overall, these findings make the relationship between smoking and AD more elusive.

This study aimed to perform Mendelian randomization (MR) analyses, using genetic variants as proxy instruments for smoking, to make causal inference between smoking and AD. As alleles are randomly allocated to offspring, MR can estimate the causal effect without bias due to confounders and reverse causation (Davey Smith and Hemani, 2014). Previous MR results in European population have reported that genetically higher smoking quantity is associated with a lower risk of AD (Ostergaard et al., 2015; Larsson et al., 2017). However, no MR studies have examined the association in East Asian population. Herein, we use the GWAS summary data in Japanese and Chinese populations through MR analysis to assess the causal relationship between smoking and AD.

## 2. Materials and methods

### 2.1. Cigarettes per day genome-wide association study data

The cigarettes per day (CPD) GWAS dataset from the Japanese population was derived from the BioBank Japan Project (BBJ) (Matoba et al., 2019).^1^ The study examined a smoking-related trait (cigarettes per day, CPD, the definition of quantity of smoking) using up to 72,655 individuals, including 58,784 males. Genotyping for the individuals was carried out using the Illumina HumanOmniExpress Exome or both the Illumina HumanOmniExpress and the HumanExome BeadChip platform. Then the study phased haplotypes and performed imputation as described (Matoba et al., 2019). BBJ have collected the medical record information from all participants, including 820 patients with dementia.^2^ According to the appendix to this CPD GWAS study, the participants included did not suffer from dementia.

### 2.2. AD genome-wide association study data

#### 2.2.1. Chinese cohort data

The AD GWAS data of the Chinese cohort were obtained from 1,500 AD case–control participants recruited by our team and performed whole genome-wide sequencing after genomic DNA extraction from peripheral blood.

##### 2.2.1.1. Inclusion criteria for participants

A total of 1,000 AD patients were included in the cohort. Patients in this study were recruited between September 2016 and September 2020 at Xiangya Hospital, Hunan Province, China. The admission of AD patients in this study followed with the “clinically probable AD” diagnostic criteria for NIA-AA diagnostic guidelines in 2011 (McKhann et al., 2011). All patients with AD were diagnosed by two or more experienced neurologists in Xiangya Hospital.

500 matched cognitive normal controls with basic information, as well as the Mini-Mental State Examination (MMSE) test, were included in this study. They come from the surrounding community of Xiangya Hospital. Written informed consent was acquired from all participants. This study was approved by the Ethics Committee of Xiangya Hospital, Central South University, Changsha, China.

##### 2.2.1.2. Whole genome-wide sequencing and GWAS analysis

Genomic DNA (gDNA) was extracted using a QIAGEN kit. The processed DNA sample was sequenced by the BGISEQ platform at the Wuhan Huada Medical Laboratory. The average sequencing depth of a single sample was not less than 30X. The original sequencing data mainly went through three steps: quality control, alignment and variant calling. PLINK v1.9 performed quality control from both individual and SNP perspectives (Purcell et al., 2007). The elimination criteria for individual-level included samples (1) are repetitive or potentially genetically related, (2) whose heterozygosity rate of SNP is not within 3 standard deviations of the mean rate, (3) with too high deletion rate of SNP loci (> 95%), and (4) report discordant phenotypic sex with X chromosomal calculated sex. The SNP-level exclusion criteria were as follows: (1) SNP loci with excessive deletion rate with a threshold of 95%, (2) SNP with sequencing depth less than 10X and genotype quality (GQ) less than 30, (3) SNP with different deletion proportion (*p* < 1 × 10^−6^) in cases and controls, (4) SNP deviating from Hardy–Weinberg equilibrium (HWE), the threshold for HWE test in this study was *p* < 1 × 10^−6^ due to the small sample size, and (5) SNP with a minor allele frequency (MAF) < 0.01 in the control group. In the end, 8,072,200 loci from 1,500 subjects passed quality control. Afterwards, we used PLINK to execute the logistic regression model between AD and control and corrected for gender, age, the first seven principal components, and *APOE* ε4 (*APOE* ε4+, *APOE* ε4−) (Purcell et al., 2007).

#### 2.2.2. Japanese cohort data

The AD GWAS of the Japanese population comprised 3,962 AD cases and 4,074 controls from the NBDC Human Database (Shigemizu et al., 2021).^3^ The AD cases were diagnosed with a probable or possible AD based on the criteria of the NIA-AA workgroups in 2011 (McKhann et al., 2011). The normal controls included subjects with normal cognition on the neuropsychological assessment. Genome-wide genotyping was performed using the Affymetrix Japonica Arrayor Affymetrix GeneChip 6.0 microarrays. Genotype imputation was conducted as detailed in the original literature (Shigemizu et al., 2021).

### 2.3. Genetic instrumental variables

In this study, we selected the instrumental variants as follows (Burgess et al., 2019): (1) SNPs associated with CPD genome-wide significantly (*p* < 5 × 10^−8^), (2) SNPs not in linkage disequilibrium (LD) (*r*^2^ > 0.001, kb = 10,000) based on the East Asian 1,000 Genomes dataset (Auton et al., 2015), (3) to exclude pleiotropy, we examined whether the SNPs were directly associated with AD in previous GWAS (*p* < 5 × 10^−8^), and (4) for SNPs not available in the outcome dataset, we searched for proxy SNPs (*r*^2^ > 0.8), using the East Asian 1,000 Genomes as a reference.

To avoid confounding factors, we examined associations between the instrumental SNPs and major risk factors of AD (low education, hypertension, middle-aged hearing loss, physical inactivity, type 2 diabetes, obesity, depression, and lack of social support) (Livingston et al., 2017). For unknown confounders, MR-Egger intercept test detected the presence of directional pleiotropy (Burgess and Thompson, 2017). Meanwhile, MR pleiotropy residual sum and outlier (MR-PRESSO) test detected the horizontal pleiotropy and outlier SNP (Verbanck et al., 2018). The significance level for the pleiotropy analyses was set as *p* < 0.05.

We calculated the proportion of variance of CPD explained by each SNP with the formula *R^2^ = 2 × EAF × (1−EAF) × (Beta/SD)^2^*. Then F-statistic was estimated using the formula *F=* $\left(\left(N-k-1\right)/k\right)\left(R^{2}/\left(1-R^{2}\;\right)\right)$ (Larsson et al., 2017). *F* > 10 could avoid weak instrument bias in MR study (Burgess et al., 2011).

### 2.4. Statistical analysis

The fixed-effect inverse-variance weighted (IVW) method was used to combine CPD associated SNPs causal estimates for AD, complementing with the weighted median (WME) function and MR-Egger regression (Burgess et al., 2019). Moreover, we conducted MR Egger intercept test, leave-one-SNP-out analysis, modified Cochran’s Q heterogeneity test, and MR PRESSO test to test the stability of the results (Lawlor et al., 2016). The funnel diagram described the reciprocal of standard error for each SNP causal effect estimate. The heterogeneity of the overall estimate was assessed based on the symmetry of the vertical lines in the funnel diagram. All statistical analyses were performed using R (v 4.0.2) and the related packages (“Two SampleMR” and “Mendelian Randomization”) (Yavorska and Burgess, 2017; Hemani et al., 2018). The significant level was set as *p* < 0.05.

## 3. Results

### 3.1. MR analysis results in the Chinese cohort

#### 3.1.1. GWAS data of genetic instrumental SNPs in the Chinese cohort

After the aforementioned screening processes, Table 1 and Supplementary Table S1 contained the summary information of the genetic instruments for the Chinese cohort. The five instrumental SNPs could account for around 0.36% of the variance of CPD. Furthermore, the calculated F-statistic was 53.

**Table 1:** Genome-wide effect information of genetic instrumental variables used in the Chinese cohort.

SNP	Cigarettes per day						AD		
	EA	Non-EA	EAF	*β*	SE	*p*	*β*	SE	*p*
rs78277894	A	G	0.336	0.040	0.006	5.63E-13	0.080	0.101	0.428
rs2435355	C	T	0.195	−0.036	0.007	3.62E-08	0.093	0.096	0.334
rs79105258	A	C	0.257	0.055	0.007	4.76E-17	−0.104	0.093	0.262
rs13329271	C	A	0.516	−0.044	0.005	2.71E-16	−0.108	0.084	0.200
rs56129017	T	C	0.260	0.131	0.006	2.18E-96	−0.122	0.088	0.164

EA, effective allele in the CPD GWAS data; EAF, effective allele frequency in the CPD GWAS data; AD, Alzheimer’s disease; SE, standard error.

#### 3.1.2. Mendelian randomization analysis in the Chinese cohort

**Figure 1:**
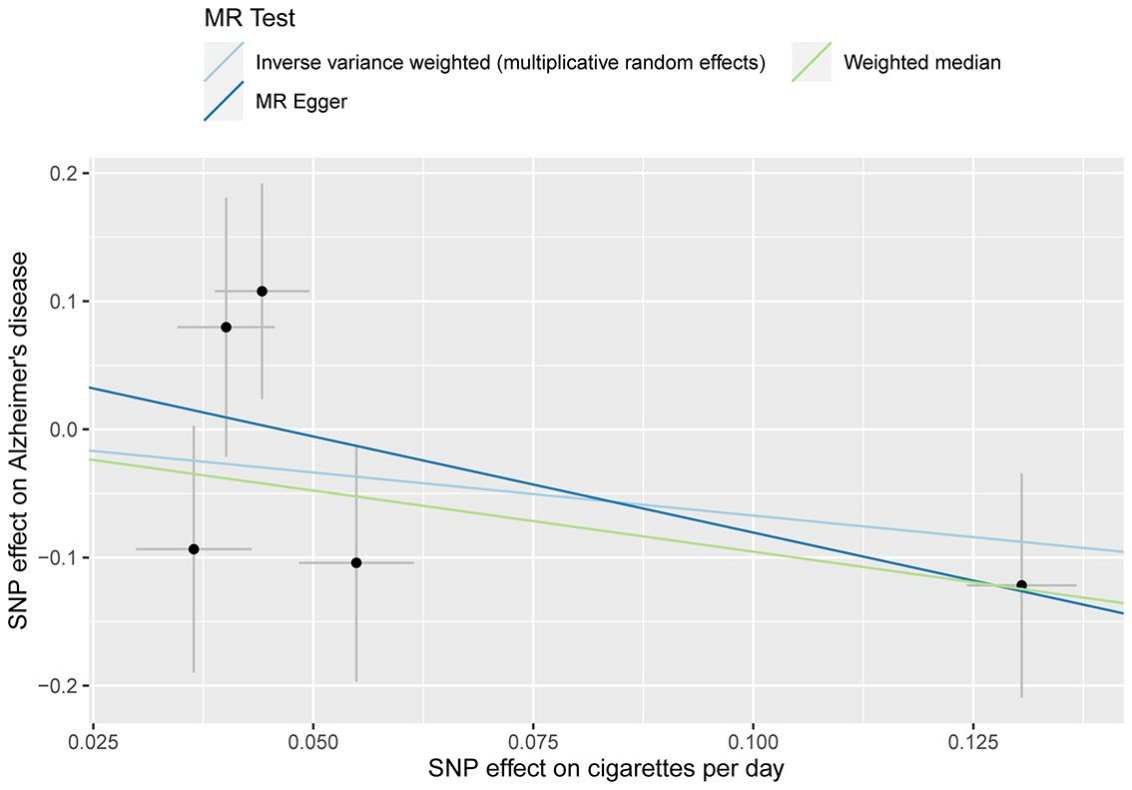
MR plots for the relationship of CPD with AD in the Chinese cohort. Analyses were conducted using the inverse-variance-weighted (IVW) MR method and complementary methods, including weighted median (WME) and MR-Egger regression approaches. Scatterplot of SNP potential effects on CPD with AD, with the slope of each line corresponding to estimated MR effect per method.

No evidence of a causal relationship between CPD and AD was discovered in the MR analysis (Figure 1 and Table 2; IVW estimate with a fixed-effects model: odds ratio (OR): 0.510, 95% confidence interval (CI): 0.149–1.749; *p* = 0.284). No horizontal pleiotropy and no SNP outliers were found by the MR-PRESSO test (*p* = 0.455). The MR-Egger intercept also indicated no directional pleiotropy (*p* = 0.531, intercept = 0.069, se = 0.098). According to Figure 1 and Table 2, the results of IVW, WME, and MR-Egger all yielded a similar pattern of effects (MR-Egger regression: *p* = 0.348, OR = 0.223, 95% CI = 0.016–3.151; WME: *p* = 0.133, OR = 0.385, 95% CI = 0.111–1.338), demonstrating the robustness of the causal associations under various MR assumptions.

**Table 2:** MR results for the relationship of CPD with AD in the Chinese cohort.

Method	OR (95% CI)	*p*	*n* SNP
Inverse variance-weighted (IVW)	0.510 (0.149–1.749)	0.284	5
MR-Egger	0.223 (0.016–3.151)	0.348	5
Weighted median estimator (WME)	0.385 (0.111–1.338)	0.133	5

The effect of CPD associated variants on AD was shown by the forest plot (Supplementary Figure S1). In the leave-one-out analysis, we found that no single SNP had an influential influence on the result (Supplementary Figure S2). With a *p*-value of 0.290 for the Q heterogeneity test, the funnel diagram (Supplementary Figure S3) revealed no discernible heterogeneity across each SNP.

### 3.2. MR analysis results in the Japanese cohort

#### 3.2.1. GWAS data of genetic instrumental SNPs in the Japanese cohort

Following the selection, Table 3 and Supplementary Table S2 displayed the summary information for the genetic instruments in the Japanese cohort. The final two SNPs were proxy SNPs: rs3825845 (pairwise *r*^2^ = 0.91) and rs12151139 (pairwise *r*^2^ = 0.95) were used to replace rs13329271 and rs56129017, respectively. SNP (rs79105258) was removed from this MR study since it was not available to find a proxy SNP in East Asian 1,000 Genomes dataset. The computed F-statistic was 56.

**Table 3:** Genome-wide effect information of genetic instrumental variables used in the Japanese cohort.

SNP	Cigarettes per day						AD		
	EA	Non-EA	EAF	*β*	SE	*p*	*β*	SE	*p*
rs78277894	A	G	0.336	0.040	0.006	5.63E-13	−0.007	0.035	0.845
rs2435355	C	T	0.195	−0.036	0.007	3.62E-08	−0.055	0.042	0.190
rs3825845	T	C	0.503	−0.041	0.005	6.95E-15	0.007	0.033	0.842
rs12151139	T	C	0.256	0.122	0.006	4.83E-92	0.018	0.038	0.636

EA, effective allele in the CPD GWAS data; EAF, effective allele frequency in the CPD GWAS data; AD, Alzheimer’s disease; SE, standard error.

#### 3.2.2. Mendelian randomization analysis in the Japanese cohort

**Figure 2:**
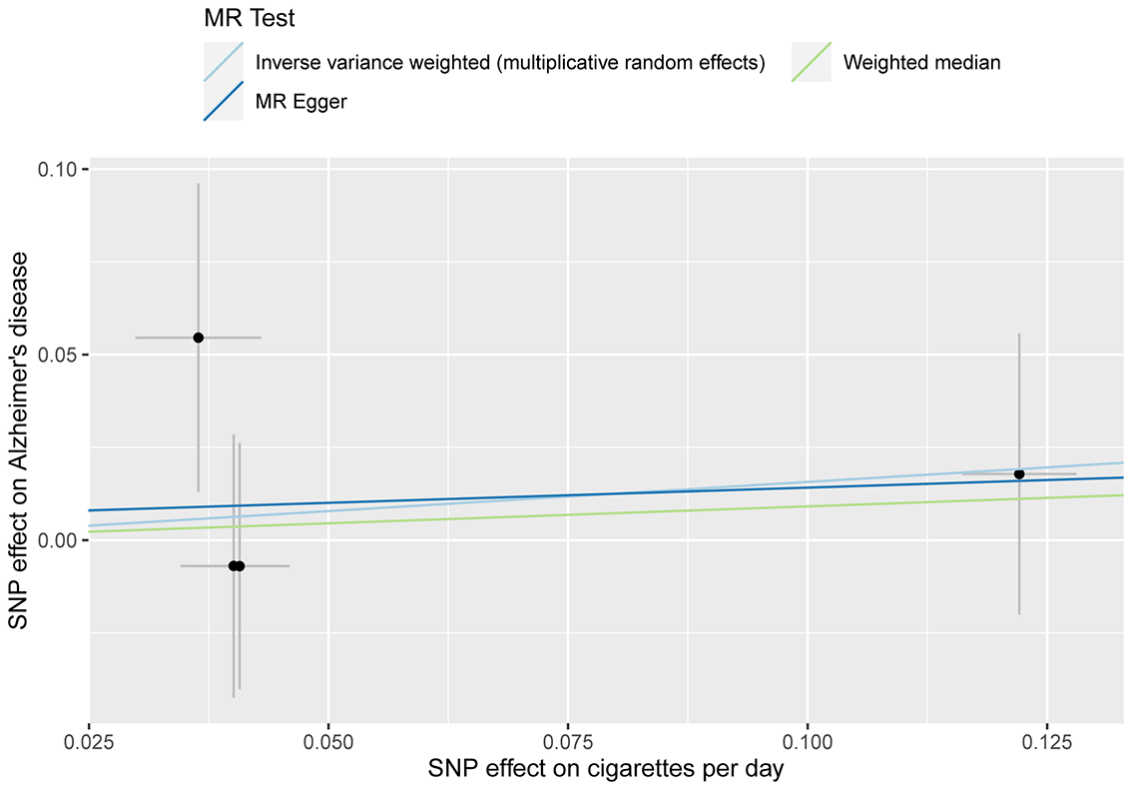
MR plots for the relationship of CPD with AD in the Japanese cohort. Analyses were conducted using different methods.

The MR analysis showed no significant association between CPD and AD (Figure 2 and Table 4; IVW estimate with a fixed-effects model: OR: 1.170, 95% CI: 0.790–1.734; *p* = 0.434). WME and MR-Egger also produced in similar results (Figure 2 and Table 4; MR-Egger regression: *p* = 0.890, OR = 1.085, 95% CI = 0.390–3.024; WME: *p* = 0.768, OR = 1.095, 95% CI = 0.390–3.024). The MR-Egger regression indicated no directional pleiotropy (*p* = 0.882, intercept = 0.006, se = 0.036). The MR-PRESSO test detected no horizontal pleiotropy (*p* = 0.802).

**Table 4:** MR results for the relationship of CPD with AD in the Japanese cohort.

Method	OR (95% CI)	*p*	*n* SNP
Inverse variance-weighted (IVW)	1.170 (0.790–1.734)	0.434	4
MR-Egger	1.085 (0.390–3.024)	0.890	4
Weighted median estimator (WME)	1.095 (0.390–3.024)	0.768	4

The forest plot was shown in Supplementary Figure S4. The leave-one-out analysis indicated that no single SNP could significantly affect the causal estimates (Supplementary Figure S5). Similarly, the funnel diagram was displayed in Supplementary Figure S6 (the *Q* heterogeneity test, *p* = 0.641).

## 4. Discussion

This study, for the first time in the Chinese and Japanese populations, found no significant association between genetically higher smoking quantity and AD risk using the two-sample MR analysis.

Our results differed from the previous meta-analyses and epidemiology studies (Xu et al., 2015; Yu et al., 2020). They indicated that smoking history increased the AD risk, especially current smoking. However, some observational studies suggested that smoking may be protective for cognitive function. A prospective study in Malaysia included 2,553 older adults aged over 60 years found that current smokers were less likely to be cognitively impaired, compared to the never smokers (Momtaz et al., 2015). In a cohort of 16,892 Chinese participants, former smokers showed better cognitive function compared to nonsmokers. Moreover, pack-years of cigarettes were positively associated with cognitive function among all participants (Ge et al., 2020). However, these results should be interpreted with caution due to the inherent drawbacks of observational studies.

In addition, some studies did not support a significant association between smoking and AD. A population-based cohort of 11,143 dementia-free individuals aged over 65 years followed up for an average of 3.8 years. Pooled results showed no significant association between smoking quantity and AD (Otuyama et al., 2020). A recent study estimated hazard ratios of 10,681 cognitively healthy adults for the transition from baseline to dementia, baseline to death, and dementia to death. Multi-state models found that smoking quantity increased the risk of death from baseline but not dementia risk or death following dementia (Johnson et al., 2021).

The contradictory findings suggested that the association between smoking quantity and AD may not be strong. MR analysis allow for a more robust inference of causal effects. In the previous MR studies, genetically predicted heavy smoking quantity associated with a lower risk of AD (Ostergaard et al., 2015; Larsson et al., 2017). Unlike our study, they were all based on European-American populations. Similarly, the two-sample MR studies both used instrumental variants located in *CHRNA3* gene of nicotine receptor gene cluster (rs1051730 in both European studies and rs13329271/rs3825845 in our study). In the European studies, rs1051730 had a nominally decreased risk of AD (*p* = 0.01) and the strongest association with higher smoking quantity. After the exclusion of the outlying SNP (rs1051730), genetically predicted smoking quantity no longer associated with AD (Larsson et al., 2017). While in our study, no instrument variant associated with AD risk. These differences confirmed that the genetic determinants with AD varied across ethnicities. Additionally, even with a larger GWAS data, the association between higher smoking quantity and lower AD risk was only suggestive significant (*p* = 0.04) in the study in 2017 (Burgess et al., 2019).

The results from this study should be interpreted in conjunction with some limitations. First of all, due to the assumption of MR analysis, the results may not apply to other ethnic groups. Second, canalization, whereby the genetic effect of smoking on AD is modified via compensatory mechanisms, may attenuate the association between genetically determined smoking quantity and AD. For instance, long-term smoking causes repeatedly upregulation and desensitization of nicotinic acetylcholine receptor (nAChR) (Xiao et al., 2020).

Despite these limitations, this study has several strengths. Firstly, MR studies mitigates the bias of observational studies, especially reverse causality and confounding factors, therefore providing a more reliable causal estimate. Secondly, the instrument variant rs13329271 used in the two cohorts is located in *CHRNA3* gene of nicotine receptor genes. Furthermore, genes where other genetic instruments situate including *CYP2A6* and *EPHX2* also relate to smoking behavior. *CYP2A6* encodes the primary enzyme responsible for nicotine metabolism, which can affect the concentration and duration of nicotine exposure in the blood, thereby associating with nicotine addiction and abstinence outcomes in smokers (Jones et al., 2022). While *EPHX2* encodes soluble epoxy hydrolase (sEH) and hydrolyzes Epoxyeicosatrienoic acids (EETs). The anti-inflammatory activity of EETs is limited after rapid hydrolysis by sEH. Inhibition of sEH reduces smoking-induced pulmonary inflammation (Li et al., 2017). Likewise, inhibition of central sEH also reduces neuroinflammation, amyloid pathology, and cognitive impairment (Ghosh et al., 2020; Grinan-Ferre et al., 2020). These functional mechanisms offered the biological justification for the association between smoking and AD and supported the high reliability of instrumental variables.

In conclusion, this MR study showed no genetic evidence of a causal relationship between CPD and AD in the Chinese and Japanese populations. Our study provided a circumspect explanation for the correlation between smoking and AD. Future MR studies should make use of larger AD GWAS data from multicenter studies in the East Asian population to determine a more robust inference for causal estimates of smoking and AD.

## Data availability statement

The datasets presented in this study can be found in online repositories. The names of the repository/repositories and accession number(s) can be found at: https://www.ncbi.nlm.nih.gov/sra, PRJNA943461.

## Ethics statement

The studies involving human participants were reviewed and approved by Ethics Committee of Xiangya Hospital, Central South University, Changsha, China. The patients/participants provided their written informed consent to participate in this study.

## Author contributions

YuZ, YG, and XX designed the experiment and analyzed the data. YuZ and YG wrote the manuscript. YuZ, YG, XX, BJ, XLia, HZ, XLiu, FQ, QP, LZ, TX, QY, SZ, ML, ZZ, and SL collected participants data. JL, BT, LS, JY, and YaZ supervised the data collection and analysis. JY and YaZ conceived the project, designed research and edited the manuscript. All authors contributed to the article and approved the submitted version.

## Funding

This study was supported by the National Key R&D Program of China (No. 2020YFC2008500), the National Major Projects in Brain Science and Brain-like Research (No. 2021ZD0201803), the National Natural Science Foundation of China (No. 81971029 and 82071216), Hu-Xiang Youth Project (No. 2021RC3028), Natural Science Foundation of Hunan Province (No. 2021JJ31134), and the Research Program on the Relationship between Nicotine and Alzheimer’s Disease [Grant No. 110201801035(JY-09)].

## Conflict of interest

The authors declare that the research was conducted in the absence of any commercial or financial relationships that could be construed as a potential conflict of interest.

The handling editor XZ declared a past co-authorship with the author BT.

## Publisher’s note

All claims expressed in this article are solely those of the authors and do not necessarily represent those of their affiliated organizations, or those of the publisher, the editors and the reviewers. Any product that may be evaluated in this article, or claim that may be made by its manufacturer, is not guaranteed or endorsed by the publisher.

## Footnotes

^1^http://jenger.riken.jp/en/result

^2^https://biobankjp.org/info/pdf/sample_collection.pdf

^3^https://humandbs.biosciencedbc.jp/hum0237-v1
