# Supplementary material for: Mendelian randomization analyses of smoking and Alzheimer’s disease in Chinese and Japanese populations
Source: Front Aging Neurosci. 2023 May 12;15:1157051. doi: 10.3389/fnagi.2023.1157051 (PMC10213305; doi:10.3389/fnagi.2023.1157051)
Supplement: Supplementary file 1 [file Data_Sheet_1.docx]

Supplementary Material

Mendelian randomization analyses of smoking and Alzheimer's disease in Chinese and Japanese populations

Yuan Zhu^†^, Ying Guan^†^, Xuewen Xiao, Bin Jiao, Xinxin Liao, Hui Zhou, Xixi Liu , Feiyan Qi, Qiyuan Peng, Lu Zhou, Tianyan Xu, Qijie Yang, Sizhe Zhang, Meng Li, Zhouhai Zhu, Sheming Lu, Jinchen Li, Beisha Tang, Lu Shen, Jianhua Yao*^,†^, Yafang Zhou*^,†^

*** Correspondence:** Jianhua Yao: [jhyao_2007@126.com](mailto:jhyao_2007@126.com); Yafang Zhou: [zyf_1981@csu.edu.cn](mailto:zyf_1981@csu.edu.cn)

# Supplementary Tables and Figures

## Supplementary Tables

**Supplementary Table 1**. Location information of genetic instrumental variables of CPD used in the Chinese cohort.

| SNP | Position（hg19） | Gene region | Exposure |
| --- | --- | --- | --- |
|  |  |  |  |
| rs78277894 | Chr8:27429192 | *EPHX2, CLU* | CPD |
| rs2435355 | Chr10:43624833 | *RET* | CPD |
| rs79105258 | Chr12:111718231 | *CUX2* | CPD |
| rs13329271 | Chr15:78914230 | *CHRNA3* | CPD |
| rs56129017 | Chr19:41416948 | *CYP2A6* | CPD |

**Supplementary Table 2.** Location information of genetic instrumental variables of CPD used in the Japanese cohort.

| SNP | Position（hg19） | Gene region | Exposure |
| --- | --- | --- | --- |
|  |  |  |  |
| rs78277894 | Chr8:27429192 | *EPHX2, CLU* | CPD |
| rs2435355 | Chr10:43624833 | *RET* | CPD |
| rs3825845 | Chr15:78910258 | *CHRNA3* | CPD |
| rs12151139 | Chr19:41433543 | *CYP2B7P* | CPD |

## Supplementary Figures


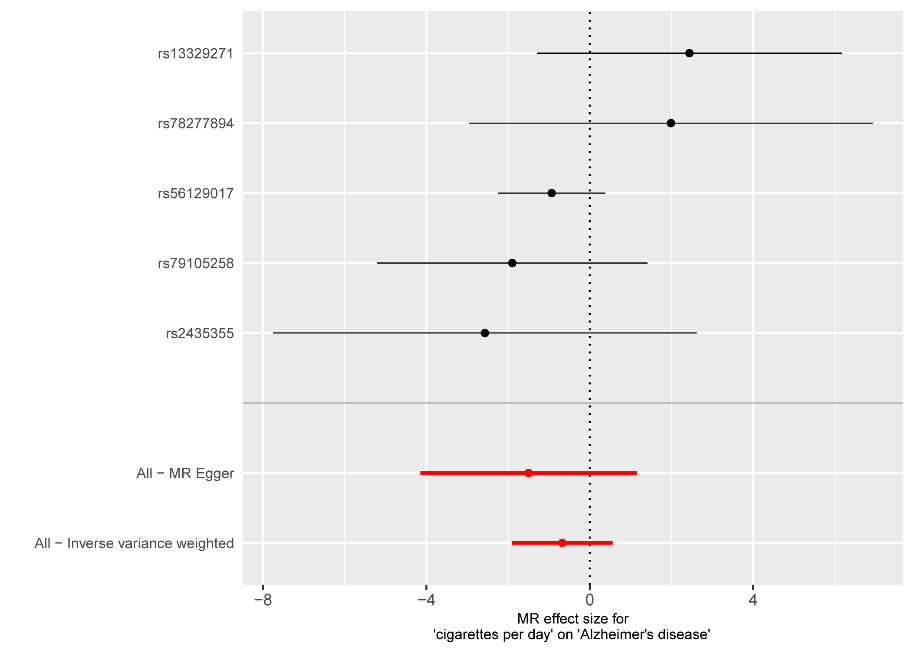


**Supplementary Figure 1.** Forest plot for genetic causal effects of CPD on AD in the Chinese cohort. The effects of CPD associated variants on AD using genome-wide association study data.


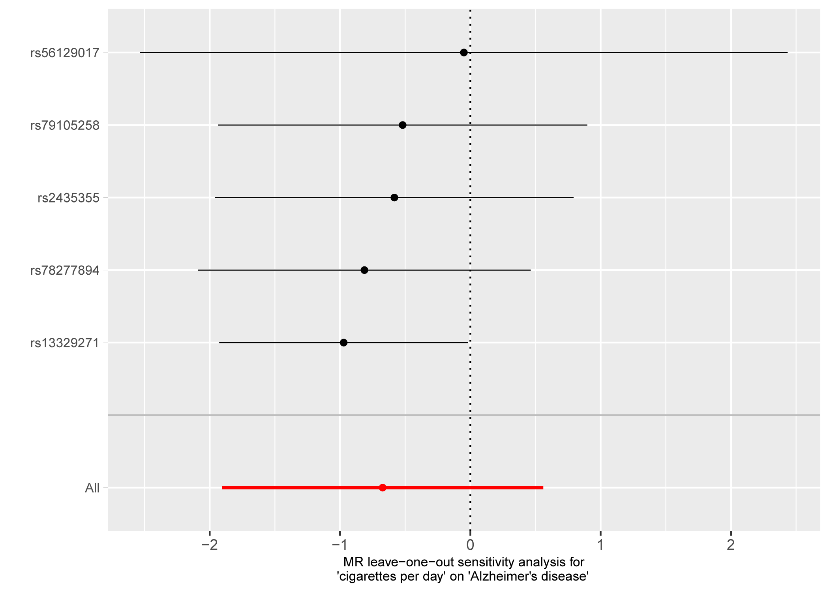


**Supplementary Figure 2.** Leave-one-out sensitivity analysis for the instrument variables used in the Chinese cohort. The solid lines represent 95% confidence intervals.


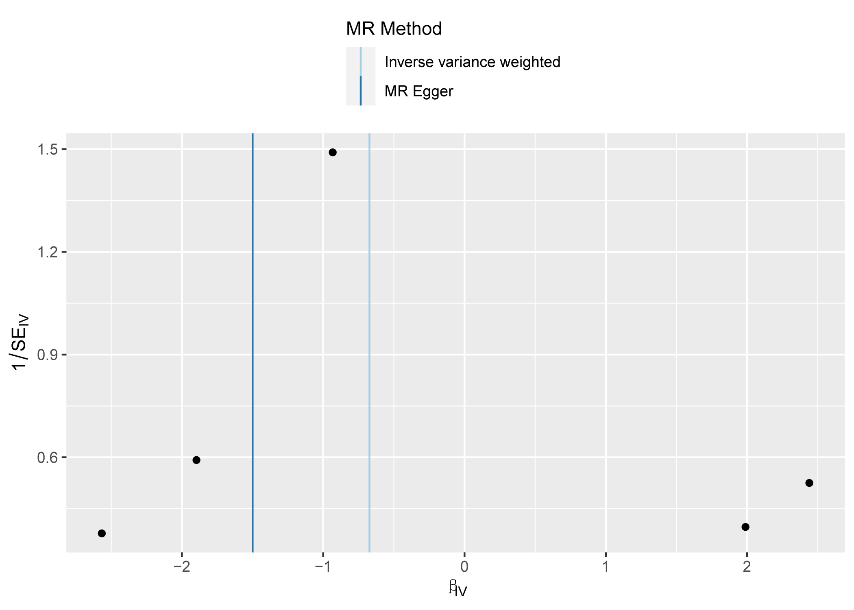


**Supplementary Figure 3.** Funnel diagram for each SNP used in the Chinese cohort.


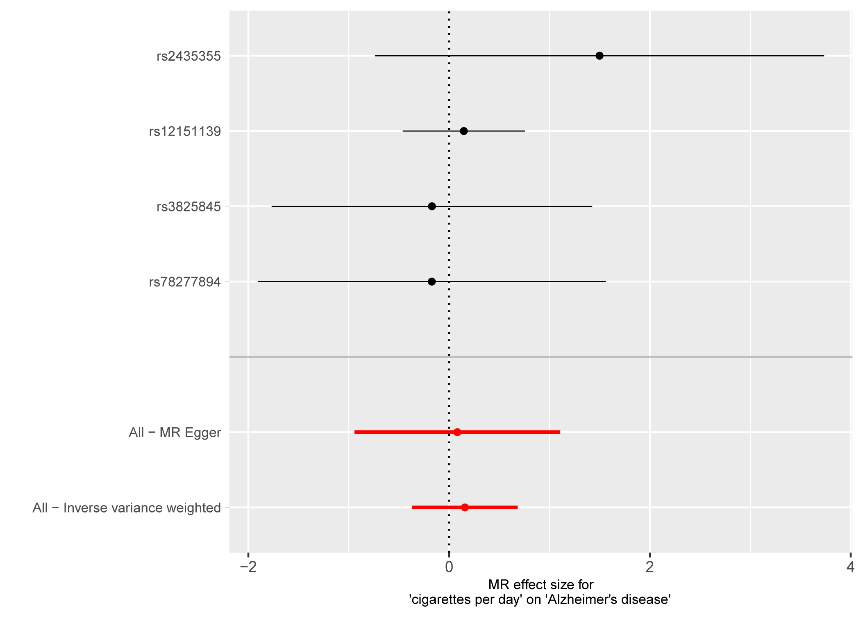


**Supplementary Figure 4**. Forest plot for genetic causal effects of CPD on AD in the Japanese cohort.


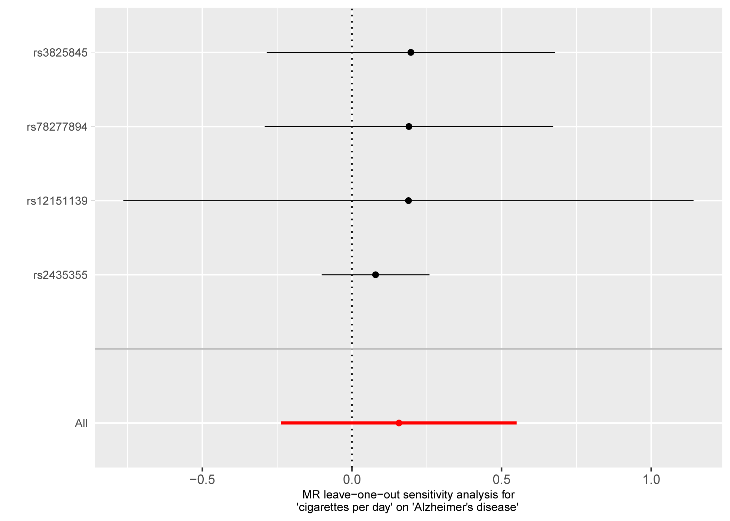


**Supplementary Figure 5**. Leave-one-out sensitivity analysis for the instrument variables used in the Japanese cohort.


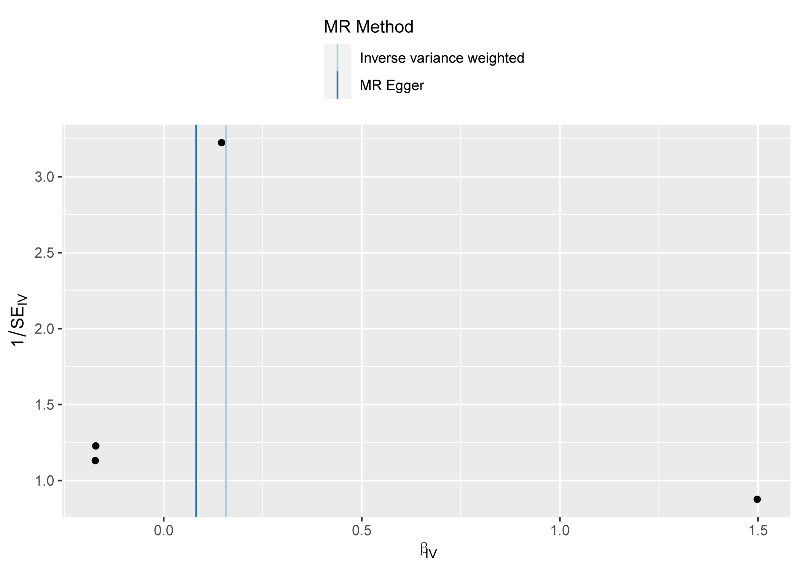


**Supplementary Figure 6.** Funnel diagram for each SNP used in the Japanese cohort
