# Supplementary figures and images for: Mendelian randomization analyses of smoking and Alzheimer’s disease in Chinese and Japanese populations
Source: Front Aging Neurosci. 2023 May 12;15:1157051. doi: 10.3389/fnagi.2023.1157051 (PMC10213305; doi:10.3389/fnagi.2023.1157051)

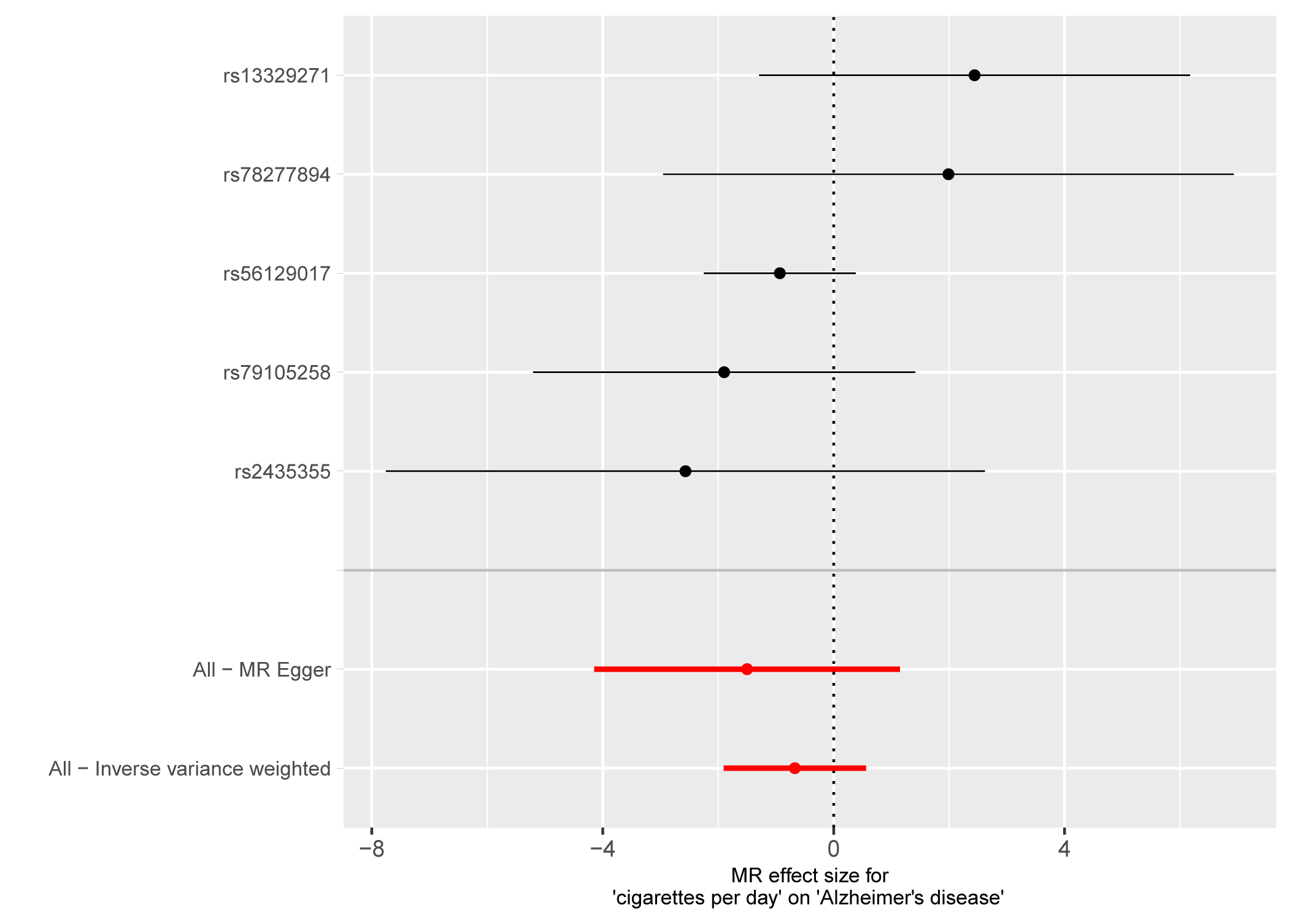

Supplement: Supplementary file 2 [file Image_1.TIF]

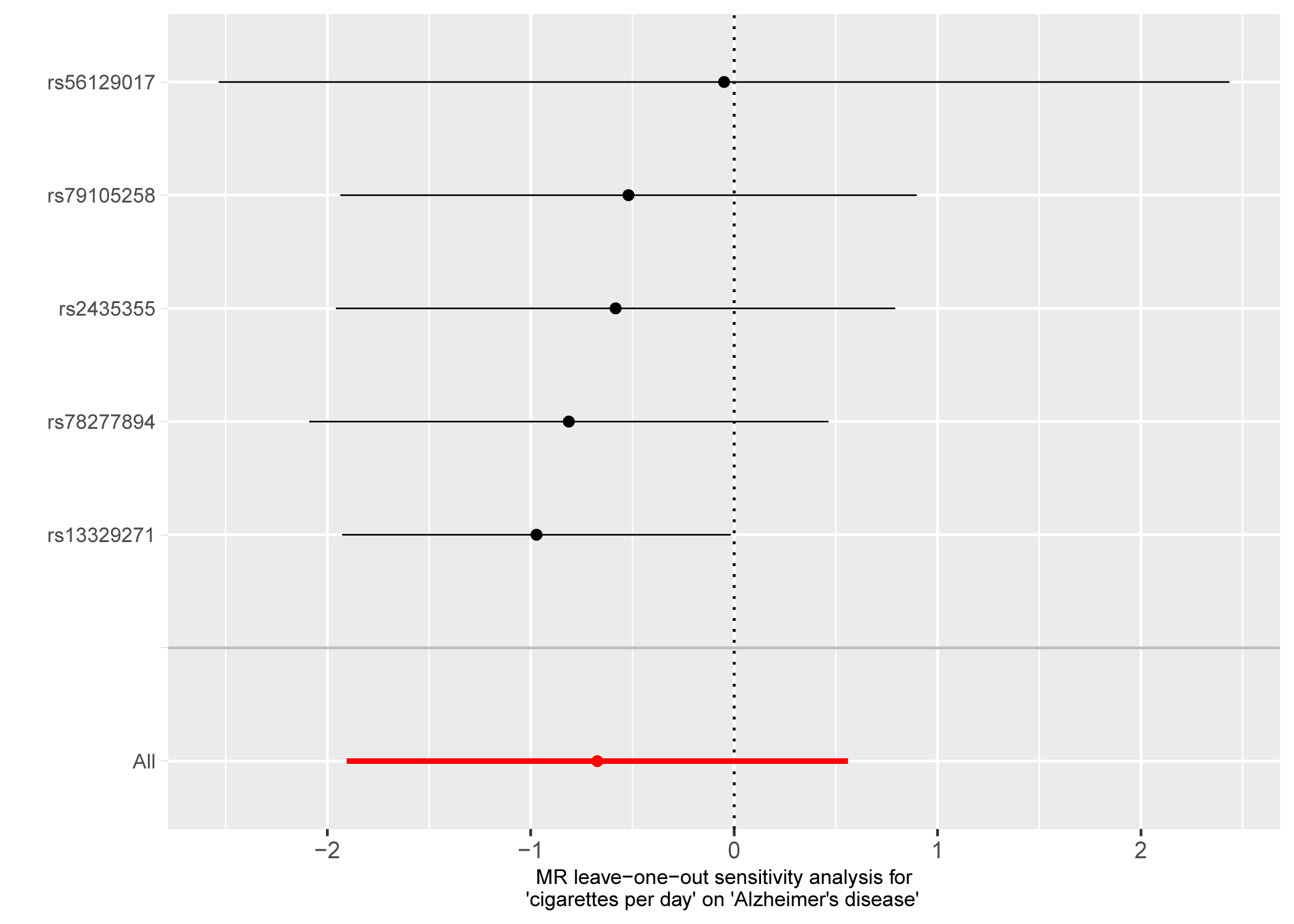

Supplement: Supplementary file 3 [file Image_2.TIF]

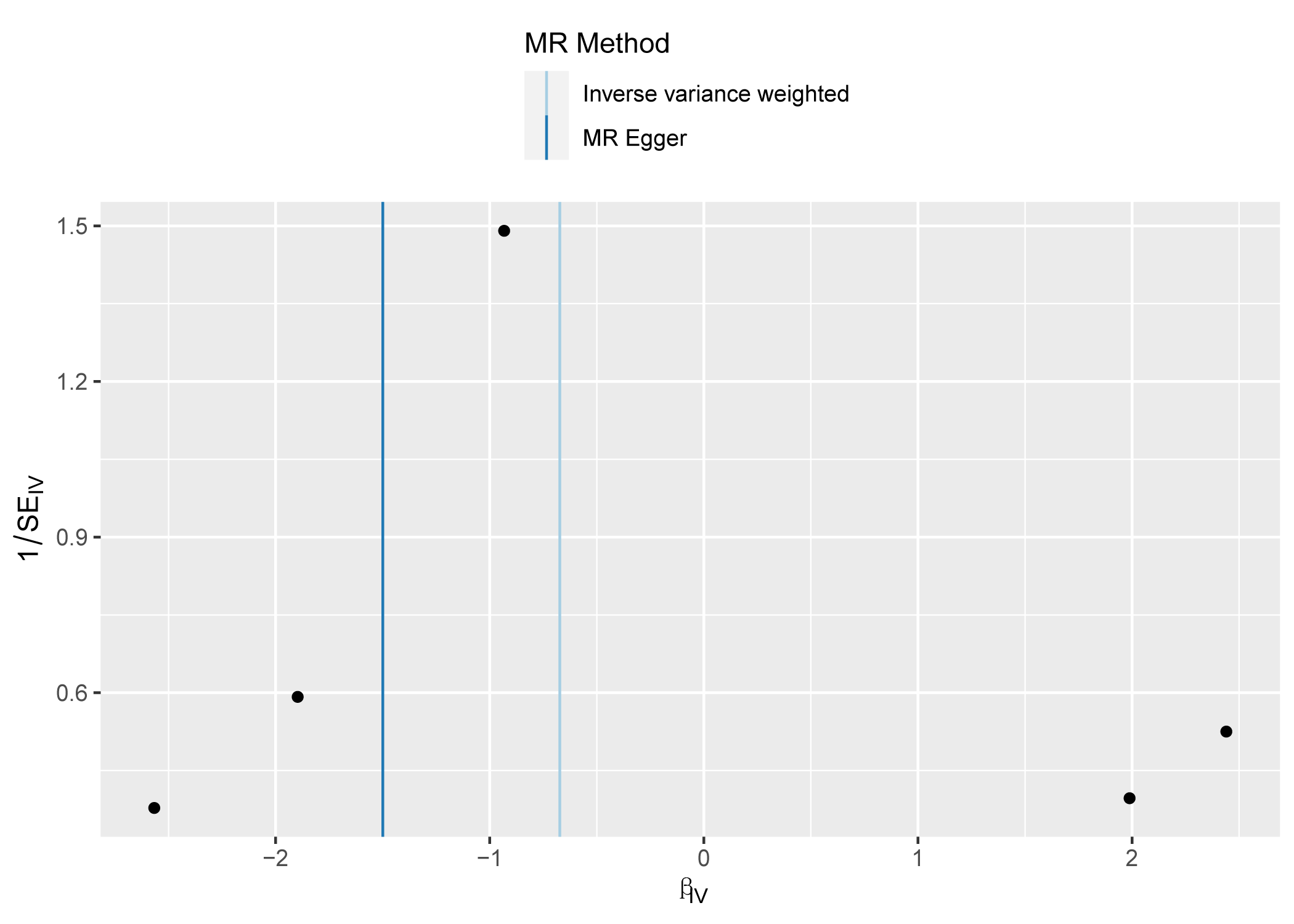

Supplement: Supplementary file 4 [file Image_3.TIF]

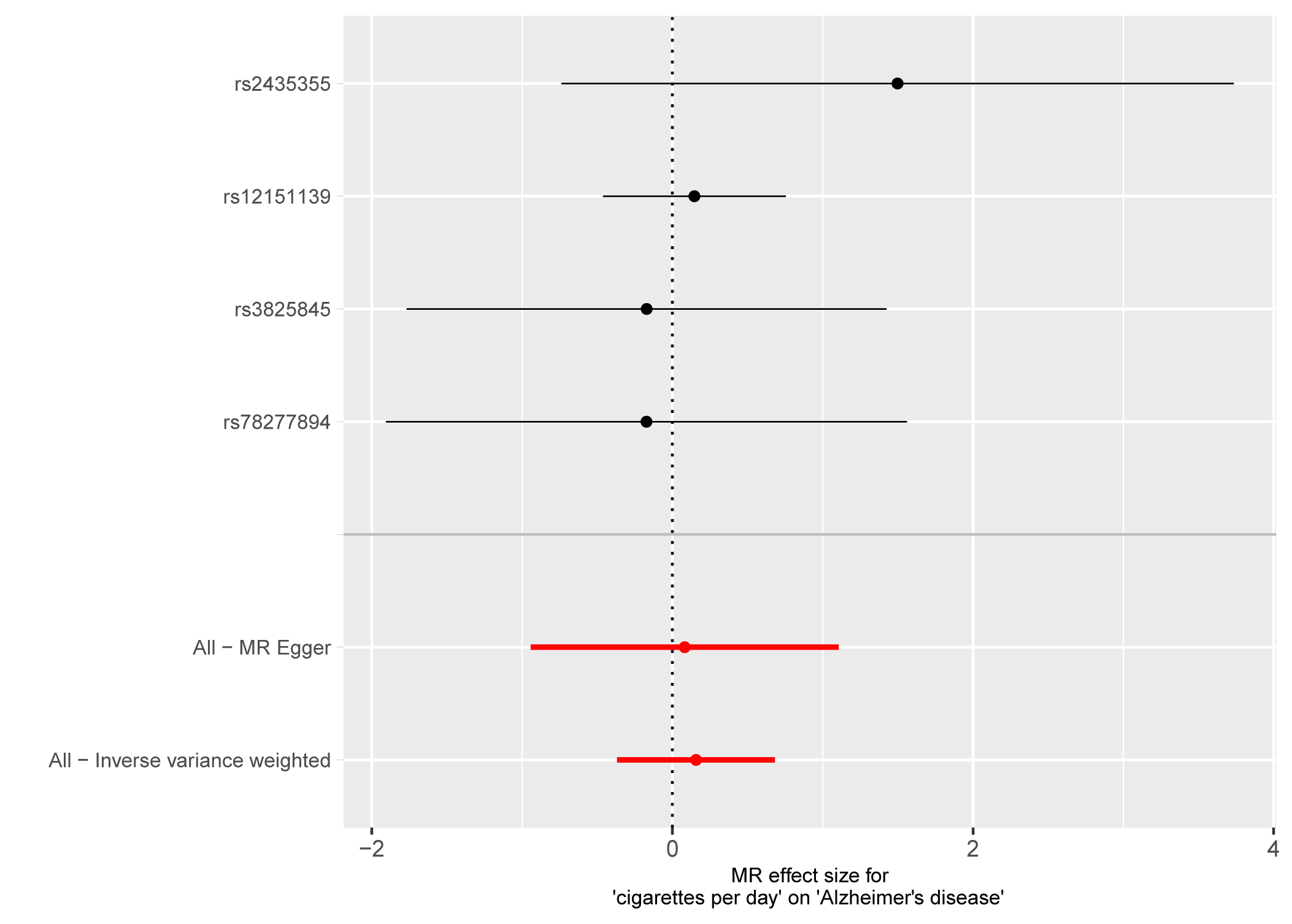

Supplement: Supplementary file 5 [file Image_4.TIF]

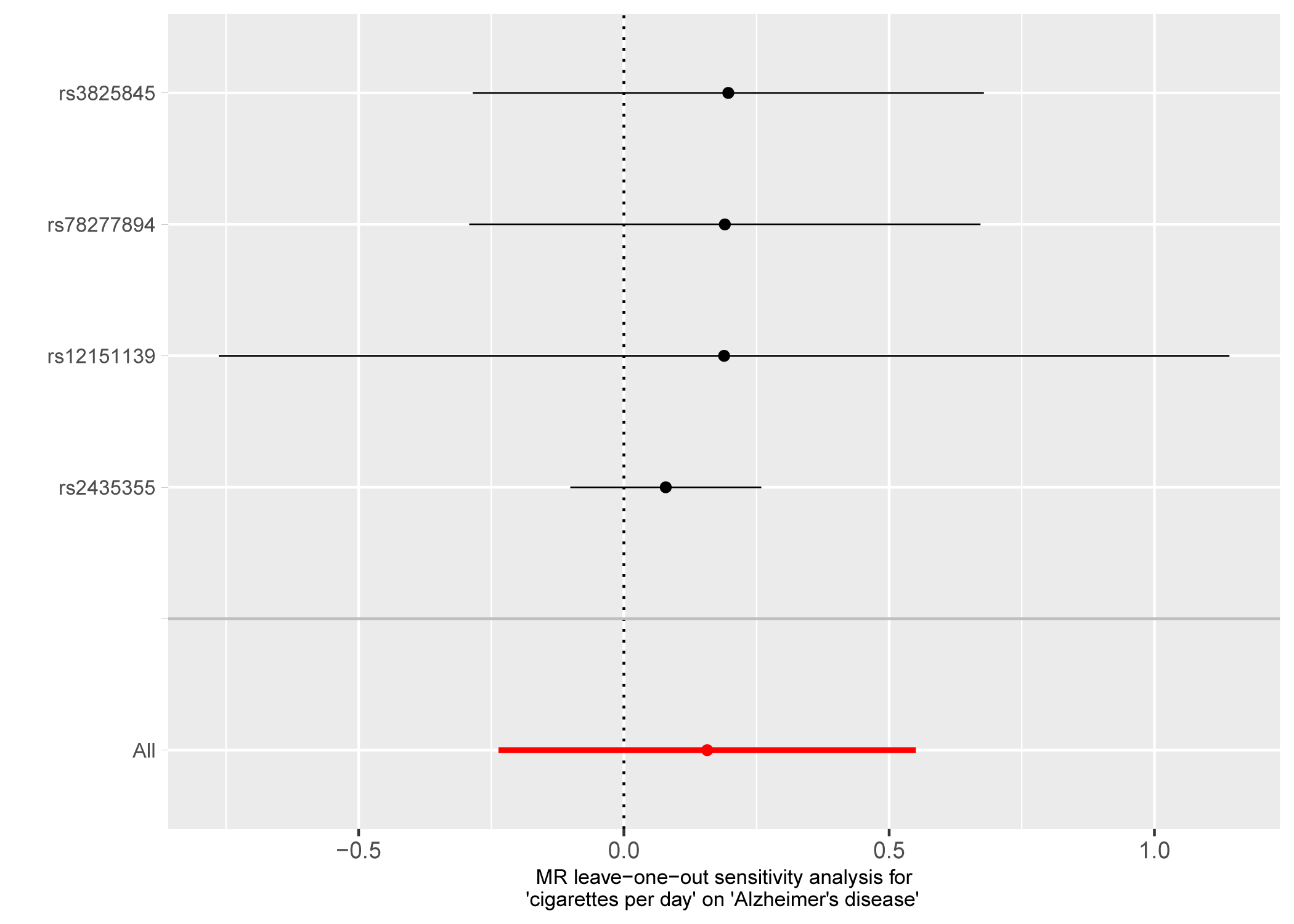

Supplement: Supplementary file 6 [file Image_5.TIF]

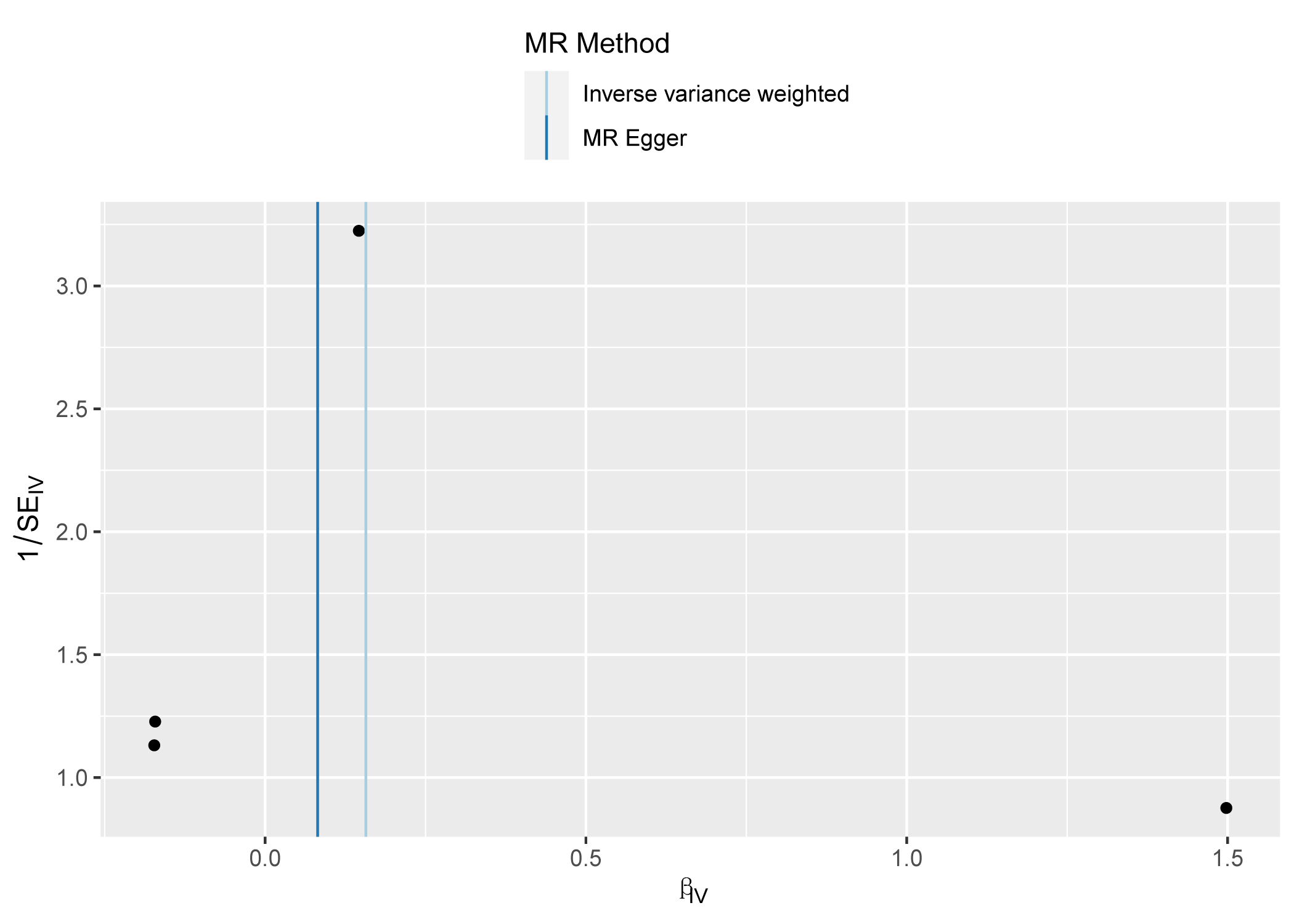

Supplement: Supplementary file 7 [file Image_6.TIF]
